# Single-Molecule Study on Histone-Like Nucleoid-Structuring Protein (H-NS) Paralogue in *Pseudomonas aeruginosa*: MvaU Bears DNA Organization Mode Similarities to MvaT

**DOI:** 10.1371/journal.pone.0112246

**Published:** 2014-11-05

**Authors:** Ricksen S. Winardhi, Sandra Castang, Simon L. Dove, Jie Yan

**Affiliations:** 1 NUS Graduate school for Integrative Sciences and Engineering, Singapore, Singapore; 2 Mechanobiology Institute, National University of Singapore, Singapore, Singapore; 3 Centre for Bioimaging Sciences, National University of Singapore, Singapore, Singapore; 4 Division of Infectious Diseases, Boston Children’s Hospital, Harvard Medical School, Boston, Massachusetts, United States of America; 5 Department of Physics, National University of Singapore, Singapore, Singapore; University of Michigan, United States of America

## Abstract

*Pseudomonas aeruginosa* contains two distinct members of H-NS family of nucleoid-structuring proteins: MvaT and MvaU. Together, these proteins bind to the same regions of the chromosome and function coordinately in the regulation of hundreds of genes. Due to their structural similarity, they can associate to form heteromeric complexes. These findings left us wondering whether they bear similar DNA binding properties that underlie their gene-silencing functions. Using single-molecule stretching and imaging experiments, we found striking similarities in the DNA organization modes of MvaU compared to the previously studied MvaT. MvaU can form protective nucleoprotein filaments that are insensitive to environmental factors, consistent with its role as a repressor of gene expression. Similar to MvaT, MvaU filament can mediate DNA bridging while excessive MvaU can cause DNA aggregation. The almost identical DNA organization modes of MvaU and MvaT explain their functional redundancy, and raise an interesting question regarding the evolutionary benefits of having multiple H-NS paralogues in the *Pseudomonas* genus.

## Introduction

The H-NS protein is an abundant nucleoid-associated protein in enteric bacteria, and plays an important role mainly in regulating gene transcription, silencing laterally acquired genes, and packaging the bacterial nucleoid [1]–[5]. The N-terminal region of H-NS forms a coiled coil structure that functions as an oligomerization domain, whereas the C-terminal region is responsible for DNA binding [6]–[9]. In *E. coli*, H-NS can associate with another H-NS-related protein called StpA, which shares 58% amino acid identity with H-NS, to form heteromeric complexes through their N-termini [1]. StpA is an H-NS paralogue that can functionally substitute for H-NS, has its own unique features, and may have distinct functions from that of H-NS [10]–[13].

**Figure 1:**
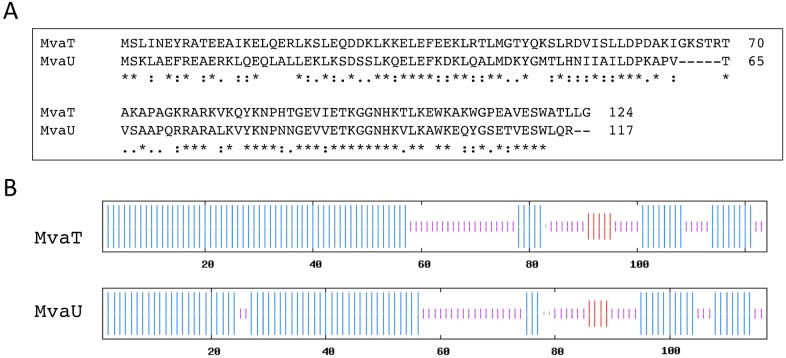
Sequence alignment and predicted secondary structure of MvaT and MvaU. (A) The sequence alignment was done with ClustalW2 [46], [47], with 46% pairwise identity. An * (asterisk) indicates positions which have a single, fully conserved residue, a : (colon) indicates conservation between groups of strongly similar properties, and a . (period) indicates conservation between groups of weakly similar properties. (B) The secondary structures were predicted using a consensus prediction method [48]. Blue vertical bars represent a-helix, red vertical bars represent extended strand, purple vertical bars represent random coil, and gray vertical bars represent ambiguous states.

H-NS paralogues also exist in other species of bacteria, some of which can functionally substitute for H-NS and complement an *E. coli* H-NS deficient mutant *in* *vivo* despite their lack of sequence similarity and identity to *E. coli* H-NS [14]–[16]. In *Pseudomonas*, MvaT and MvaU have been identified as the paralogues of H-NS that share structural and functional similarity to H-NS despite sharing <20% sequence identity to H-NS [16] (see Figure 1 for sequence alignment and predicted structural elements of MvaT and MvaU). Transcriptional profiling using DNA microarrays revealed that MvaT from *Pseudomonas aeruginosa* regulates the expression of hundreds of genes [17]–[19]. Moreover, MvaU has been shown to function coordinately with MvaT, occupying the same regions of the chromosome and co-regulating the expression of ∼350 genes [20]. The amount of genes they regulate is similar to that regulated by H-NS and StpA in E. coli, which have intracellular concentrations of few micromolar [21]. Therefore, we reason that MvaT and MvaU have similar intracellular concentrations. The deletion of either MvaT or MvaU leads to the increase in production of the other, indicating cross-regulation of the two proteins and that they can functionally compensate each other [22]. In addition, MvaT and MvaU show a binding preference for AT-rich regions of the chromosome, suggesting that these proteins are involved in xenogeneic DNA silencing [20]. This preferential binding to AT-rich regions seems to be shared among H-NS family proteins [23]–[26]. Other than the AT-rich preferential binding, in general H-NS family proteins bind DNA non-specifically, which is consistent with their role as abundant nucleoid associated proteins that play a crucial role in organizing chromosomes.

MvaT and MvaU are thought to be structurally similar to H-NS: the DNA binding domain located in the C-terminus of MvaT and MvaU is more conserved compared to H-NS, while their multimerization domain located in the N-terminus is more divergent [14]. It has been suggested that MvaT and MvaU are the functional counterparts of H-NS and StpA, as they have reciprocal regulatory interaction and form heteromeric complexes through their oligomerization domain in the N-terminus [22]. The majority of MvaU in the cell may be associated with MvaT as heteromeric complexes due to its low copy number compared to MvaT [22].

The molecular mechanism of how nucleoid-associated proteins organize DNA (i.e. DNA organization mode) may be related to how they perform their *in* *vivo* function. For example, the mechanism of gene silencing by H-NS and its anti-silencing activity are largely explained based on its DNA organization modes [27]–[30]. Previous AFM studies reported that MvaT can induce DNA bridging [31]. However, our recent study has revealed that MvaT can form rigid nucleoprotein filaments on double-stranded DNA, which can mediate DNA-bridging and form compact DNA structures at high protein concentrations [32]. In our single-molecule DNase1 cleavage assay, we found that formation of MvaT nucleoprotein filaments can protect DNA from DNase1 cleavage, while negligible protection was found when we used higher-order oligomerization defective mutants that failed to form nucleoprotein filaments (unpublished data). Since the MvaT mutants failed in both repressing *cupA* gene expression *in* *vivo* and forming nucleoprotein filaments [32], [33], these data further support the relevance of filaments formation to the role of MvaT as a gene silencer. Filament formation may be a conserved property of H-NS-like gene-silencing proteins across prokaryotes, as it is widely found in StpA from *E. coli*, MvaT from *Pseudomonas aeruginosa*, Lsr2 from *Mycobacterium tuberculosis*, and Alba1 from *Sulfolobus solfataricus* [32], [34]–[36]. Mutations that cause loss of gene-silencing function have been shown to disrupt filament formation by MvaT and H-NS [28], [32], suggesting that the formation of nucleoprotein filaments is essential for H-NS-like proteins to perform their gene silencing functions.

In spite of its importance as the paralogue of MvaT in the *Pseudomonas* genus, the binding properties of MvaU to DNA are much less well understood. Although MvaT and MvaU show some degree of functional redundancy, it does not necessarily mean that they have identical DNA binding properties. For instance, StpA, the paralogue of H-NS in *E. coli*, can cause DNA bridging while the nucleoprotein filament formed by StpA is insensitive to KCl concentration, temperature, and pH, unlike the nucleoprotein filament formed by H-NS [34]. This raises the question on the organization modes of MvaU to DNA, which may be relevant to how MvaU performs its *in* *vivo* function as the paralogue and binding partner of MvaT. In this work, we elucidate the binding properties of MvaU to DNA, and the conformations of MvaU-DNA complexes, which has implication on how gene silencing may be achieved.

## Materials and Methods

### Proteins

Plasmid pET24-MvaU-His6 was transformed into *E. coli* strain BL21 (DE3). Transformed cells were grown at 37°C in LB medium with 50 µg/mL of kanamycin. At OD_600_ of 0.6, the cells were induced with 1 mM IPTG for 4 hours at 37°C. The cells were harvested and resuspended in cell lytic solution for 2 hours on ice and centrifuged for 30 minutes at 10,000 r.p.m. to collect the supernatant. 6xHis-tagged MvaU was purified by gravity-flow chromatography. Nickel-charged resin (Ni-NTA Agarose, Qiagen, Singapore) was added to the supernatant and incubated for 2 hours on ice, and the sample was loaded on the purification column. The pelleted resin was washed several times with 50 mM potassium phosphate buffer (pH 8.0), 1 M NaCl, 25 mM Imidazole, before eluted with 50 mM potassium phosphate buffer (pH 8.0), 500 mM NaCl, 250 mM Imidazole. Protein purity and identity were verified by SDS-PAGE and mass spectrometry, and the concentration was measured using Nanodrop ND1000 (Wilmington, USA), with an extinction coefficient of 15,470 M^−1^cm^−1^. Glycerol was added to the stock protein (40–50%) and the protein was stored at −20°C.

### Transverse Magnetic Tweezers

λ-DNA labelled with biotin on both ends were stretched between streptavidin-coated cover slip and streptavidin-coated paramagnetic bead as described previously [32]. The position of the magnet was adjusted to control the amount of force applied on the DNA and the extension of DNA at each force value was recorded in real time using LabVIEW software. We performed force-scanning procedure as follows. The DNA was initially held at high force (∼10 pN), and the force was reduced successively to several lower forces down to ∼0.1 pN. The force-extension curves measured during decreasing force are referred to as force-decrease curves. Following the force-decrease scan, the force was increased successively at the same set of forces to obtain force-increase curves. Overlapping force-decrease and force-increase curves indicates that MvaU-mediated DNA organization has reached a steady state. If protein-DNA interaction is not at equilibrium, hysteresis between the two curves is expected. In the measurements, the DNA was held for ∼30 seconds at each force and the extension of the DNA at a certain force was obtained from the average values of the last 15 seconds. Solid lines were drawn connecting the data points to guide the eye. The stretching experiments were done in 50 mM KCl, 10 mM Tris pH 7.5 at room temperature, unless otherwise stated, while varying the protein concentration. To determine the effect of protein binding on single DNA, for each condition the data presented on the manuscript is a representative experiment performed on the same DNA tether. The general trend is reproducible and consistent in multiple independent experiments (see Figure S1 for additional data obtained from independent experiments).

### Atomic Force Microscopy

We used glutaraldehyde-functionalized mica to immobilize protein-DNA complexes for imaging experiments, as described previously [32]. 0.2 ng/µL of linearized ΦX174 were incubated with various amounts of MvaU in 50 mM KCl, 10 mM Tris pH 7.5 at room temperature for 1 minute before the sample was deposited on the glutaraldehyde-functionalized mica for 15 minutes. The mica was gently rinsed with deionized water and dried with nitrogen gas. Images were acquired on a Molecular Imaging 5500 AFM (Molecular Imaging, Agilent Technologies) using tapping mode with silicon cantilever (Photonitech, Singapore).

## Results

### MvaU binding causes DNA stiffening and DNA folding

**Figure 2:**
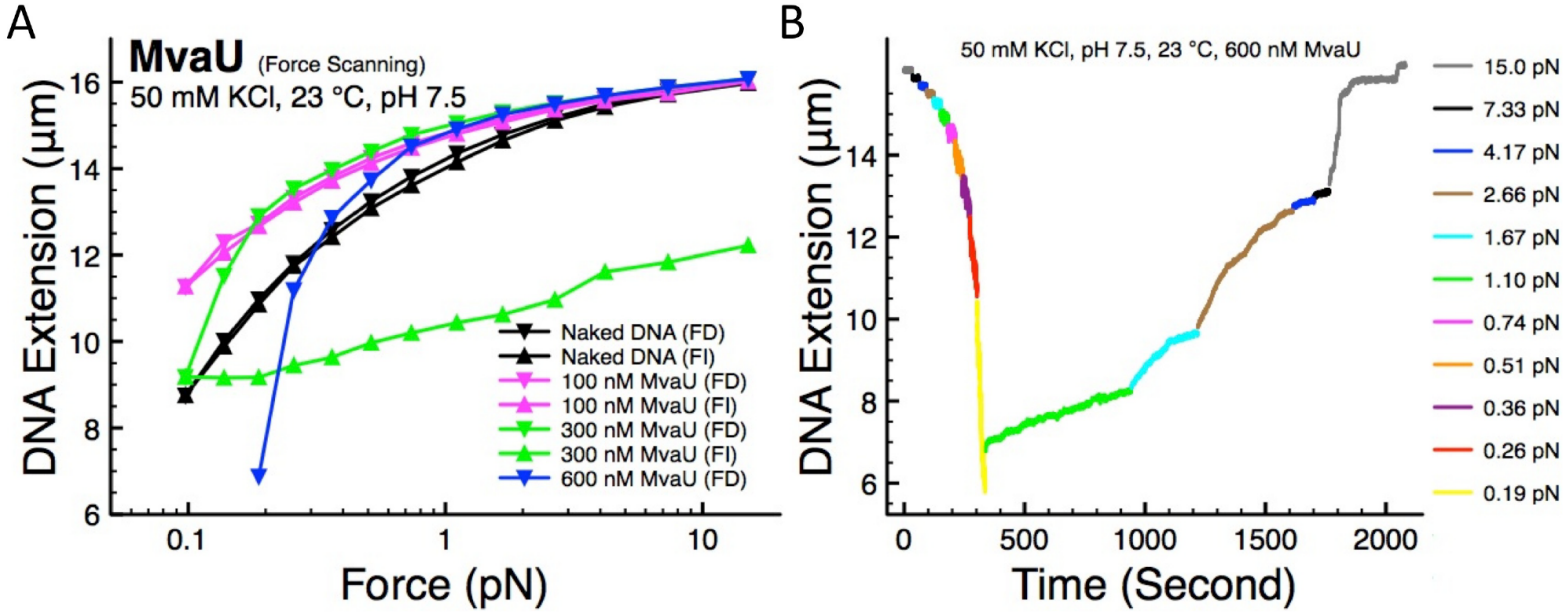
Single-molecule stretching experiments on MvaU-DNA. (A) In the presence of MvaU, the DNA was stiffened as indicated by higher extension compared to naked DNA at the same force. Along with DNA stiffening, increase in DNA folding at higher concentration was also observed, indicated by either hysteresis or DNA folding. The DNA folding at 600 nM MvaU occurred at higher force, and the force-increase scan was not recorded to prevent the DNA extension from dropping below the minimal measurable extension. (B) The time-course data of the stretching experiment in panel A, showing the dynamics of force-decrease that leads to progressive DNA folding, followed by force-increase that gradually unfold the MvaU-DNA.

To elucidate the molecular mechanism of how MvaU organize DNA (i.e. the DNA organization mode of MvaU) we performed stretching experiments on single λ-DNA molecules using force-scanning procedure as detailed in the Methods section. The force-extension curves of DNA incubated with various MvaU concentrations in 50 mM KCl, pH 7.5, at 23°C were investigated (Figure 2A). In the absence of MvaU, the force-decrease and force-increase curves of the naked DNA overlap each other, as expected for stretched molecules in equilibrium (Figure 2A, black data). In the presence of 100 nM MvaU (Figure 2A, magenta data), the DNA is stiffened as indicated by the higher extension compared to naked DNA at the lower force regime. At the high force regime, the curves nearly overlap with the naked DNA curves, showing that the nucleoprotein contour length remains unaffected. We observe no hysteresis between the force-decrease and force-increase curves, indicating that the protein binding and the resulting DNA stiffening have reached a steady state.

As the MvaU concentration in the buffer was increased to 300 nM (Figure 2A, green data), the force-decrease curve shows a similar level of DNA stiffening compared to 100 nM MvaU, indicating a saturation of the stiffening effect. The force-increase curve, however, does not overlap the force-decrease curve and falls below the naked DNA curve. The non-overlapping curves indicate a non-equilibrium state of the protein-DNA complexes while the lower extension of the force-increase curve indicates protein-induced DNA folding that results in the apparent hysteresis. However, the conformation of nucleoprotein complexes that corresponds to the observed protein-induced DNA folding cannot be distinguished in our stretching experiments. Further increase of the MvaU concentration to 600 nM results in a similar level of DNA stiffening at forces >0.8 pN (Figure 2A, blue data). At forces <0.2 pN, the DNA extension dropped below the extension of naked DNA due to DNA folding. The extension data at forces <0.2 pN and the force-increase curve were not recorded to prevent the DNA from being folded below the minimum extension that can be recorded by our instrument (∼2 µm). The occurrence of DNA stiffening at higher force prior to DNA folding at lower force in the presence of 600 nM MvaU suggests that the DNA organization mode of MvaU can be regulated by force. In addition, the DNA folding is enhanced when the MvaU concentration is increased.

In another independent experiment, the time-course data of MvaU-induced DNA folding and unfolding process in the presence of 600 nM MvaU is plotted in Figure 2B. In this experiment, the folding occurred at forces <0.51 pN and the extension dropped to ∼6 µm, before the force was increased to 1.1 pN to stop the folding. Forces up to 15 pN are required to completely unfold MvaU-DNA to its initial extension. Since DNA folding and unfolding are non-equilibrium in nature, the magnitude of folding and unfolding force may vary across experiments (see Figure S1B for another independent time-course experiment performed at identical conditions). Due to this, we do not use the level of hysteresis as an absolute measure of DNA folding.

### MvaU organizes DNA into bridges and compact structures in a concentration-dependent manner

**Figure 3:**
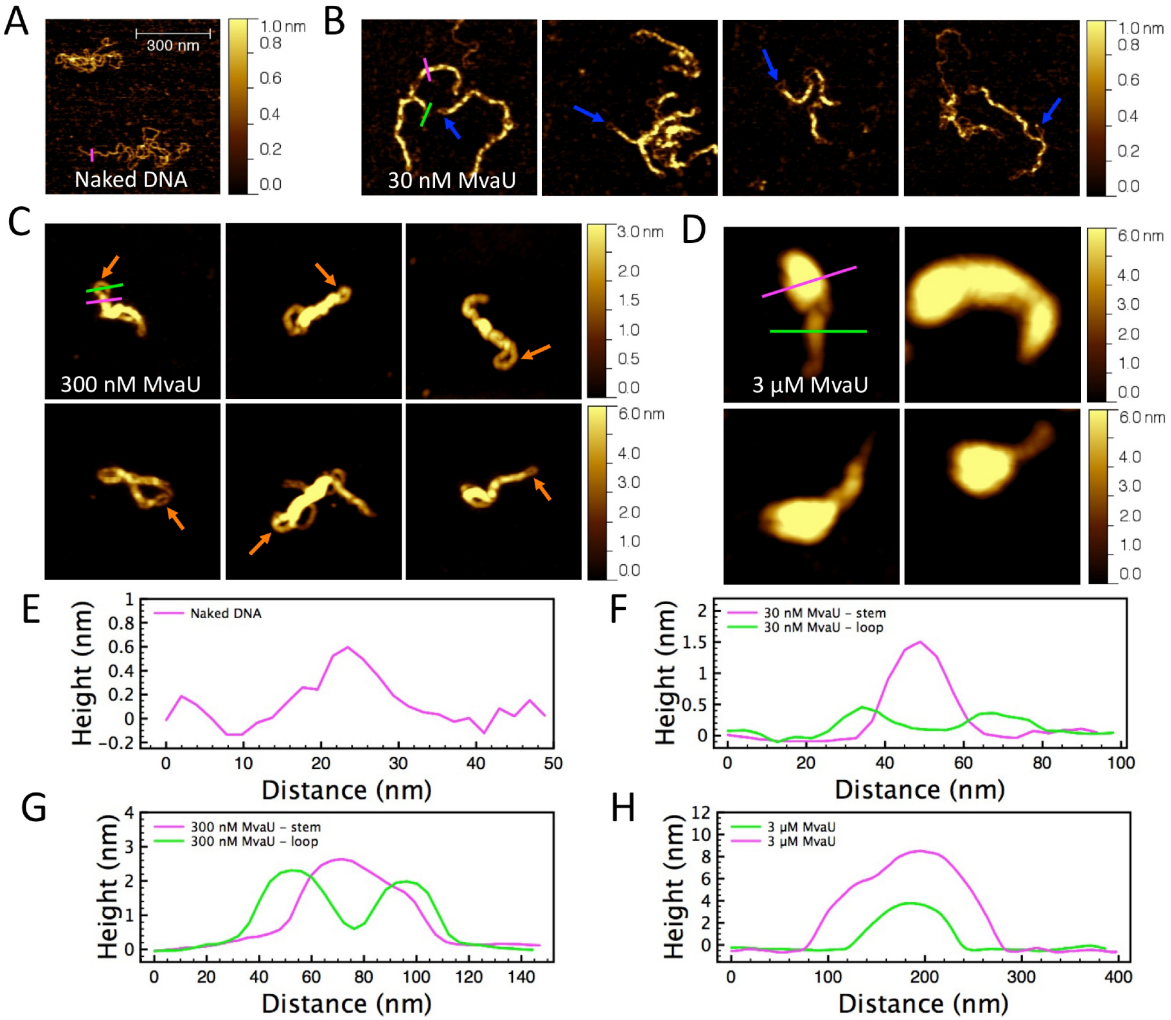
The conformations of MvaU-DNA at various protein concentrations in AFM imaging experiments. (A) Naked ΦX174 DNA adopting random coiled structure. (B–D) ΦX174 DNA complexed with 30 nM (B), 300 nM (C), and 3 µM (D) MvaU. Blue arrows in panel B indicate small uncoated DNA loops at the end of the DNA bridges. The orange arrows in panel D show large bridges formed through further association or compaction of thinner MvaU nucleoprotein filaments. (E–H) Line profiles of the loop end structures as indicated by the magenta and green lines in panel A–D. The surface area for all the images are 0.7 µm×0.7 µm.

The results of single DNA stretching experiments give us information on the impact of protein binding on DNA mechanical response. To complement these results, we performed AFM imaging experiments to study the conformations of MvaU-DNA formed at various protein concentrations. ΦX174 DNA was used for the imaging studies because of its convenient size (5,386 bp) for AFM imaging. In the absence of MvaU, the DNA assumed a random-coiled structure in solution (Figure 3A). At 30 nM MvaU (corresponding to 1 MvaU monomer for every 10 bp), the DNA was predominantly organized into DNA bridges that assume extended conformations (Figure 3B). We define DNA bridges as two DNA segments bridged by a track of proteins. The two DNA segments can be bridged from within the same DNA molecule through DNA looping or from different DNA molecule. The blue arrows in Figure 3B indicate small loops formed at the end of the DNA bridges. These end-loops are also observed previously for H-NS-DNA bridging, which we believe is caused by naked DNA segment that loops back and associate with MvaU-coated DNA segments of the same molecule [37]. The resulting sharp DNA curvature inside the loop is often unassociated with MvaU, which can be explained by a higher bending energy cost to form a curved MvaU nucleoprotein filament, which has an increased bending rigidity compared to naked DNA. This can hinder further MvaU binding, causing the naked DNA loops that we observe. Representative line profiles of naked DNA as well as loops and stems of the DNA bridges are shown in Figure 3E–F. The measured height of the loops is 0.37±0.08 nm, which is similar in comparison to 0.55±0.11 nm obtained for naked DNA, indicating that the loops of the DNA bridges are uncoated DNA molecule. The height obtained for naked DNA is lower than the expected dsDNA thickness of ∼2 nm, which is a well-known anomaly in tapping mode AFM caused by adhesion at the sample surface [38], [39]. In comparison, the stem of the DNA bridges has a measured height of 1.20±0.18 nm.

At a higher MvaU concentration of 300 nM (corresponding to 1 MvaU monomer for every bp), higher-order rod-like structures were observed along with compact nucleoprotein structures. The orange arrows indicate large loops formed at the end of the higher-order rod-like structures. Representative line profiles of the loops and stems of the rod-like structures are shown in Figure 3G. The height of the large loops is 2.03±0.44 nm, suggesting that they are coated by proteins. These loops are considerably larger than the stem of DNA bridges formed in 30 nM MvaU, suggesting that DNA inside the loop is organized into DNA bridges or higher order structures. The stem of these rod-like structures have a measured height of 4.30±1.32 nm. The nucleoprotein complexes vary considerably in size, suggesting that they are likely formed by adsorption of additional DNA or DNA-protein complexes to simple DNA bridges along the stem direction. Note that higher-order structures are also observed previously for H-NS-DNA complexes [37].

At 3 µM MvaU (corresponding to 10 MvaU monomers for every bp), we only found large and compact rod-like nucleoprotein complexes (Figure 3D). These structures are considerably larger with height generally >4 nm, which can still be explained by adsorption of additional DNA or DNA-protein complexes along the stem direction. Representative line profiles of the large rod-like structures formed at 3 µM MvaU are shown in Figure 3H. The increasing occurrence and size of the large rod-like structures as MvaU concentration was increased can be explained by free MvaU in solution that associates with nearby nucleoprotein filaments to form larger nucleoprotein structures (see Discussion for more details).

In general, our AFM data agree with our observation on the magnetic tweezers experiments. The apparent DNA stiffening suggests that the first step of MvaU binding is an assembly of a rigid nucleoprotein filament, which can mediate DNA bridging when it meets a naked DNA molecule to form extended DNA bridges as seen in AFM (Figure 3B). A higher MvaU concentration leads to DNA folding in single-DNA stretching experiments and formation of large rod-like structures in AFM imaging experiments. This higher-order DNA organization is similar to the nucleoprotein structures formed by MvaT and Lsr2 [32], [35]. We propose that these observations are related to a concentration-dependent distribution of MvaU oligomerization states [22], [33], [40], which is elaborated upon in the Discussion.

### Dependence of MvaU-DNA binding properties on environmental factors

Environmental factors, such as KCl concentration, pH, temperature, and MgCl_2_ concentration, have been found to significantly affect the DNA organization modes of nucleoid-associated proteins such as H-NS, StpA, and IHF, and moderately affect DNA binding of MvaT and Lsr2 in physiological conditions [32], [34], [35], [41], [42]. The responses of protein-DNA complexes to environmental cues may be important to their roles *in* *vivo*. Thus, we would like to determine the impact of KCl concentration, pH, temperature, and MgCl_2_ concentration on MvaU-DNA. The elasticity of naked DNA is insensitive to variation in these factors, thus we can attribute any observed changes in force-extension curve to changes in protein-DNA interaction (see Figure S2 for naked DNA control).

We fixed the concentration of MvaU to 300 nM while KCl concentration, pH, temperature, or MgCl_2_ concentration was varied. Using the force-scanning procedure, we are able to gain rich insights on the impact of these environmental factors to MvaU-induced DNA stiffening and DNA folding. The level of DNA stiffening can be assessed based on the force-decrease curves at higher force values (>1 pN), as MvaU folding may interfere with DNA extension measurement at lower force values (<1 pN). In addition, the level of DNA folding is based on the observation of hysteresis between the force-decrease and force-increase curves, i.e. protein binding have not reached a steady state due to the non-equilibrium nature of DNA folding. As the level of hysteresis may vary from one experiment to another due to the non-equilibrium nature of DNA folding, we only assess the general trend of increased or decreased level of DNA folding in response to variation in solution conditions.

**Figure 4:**
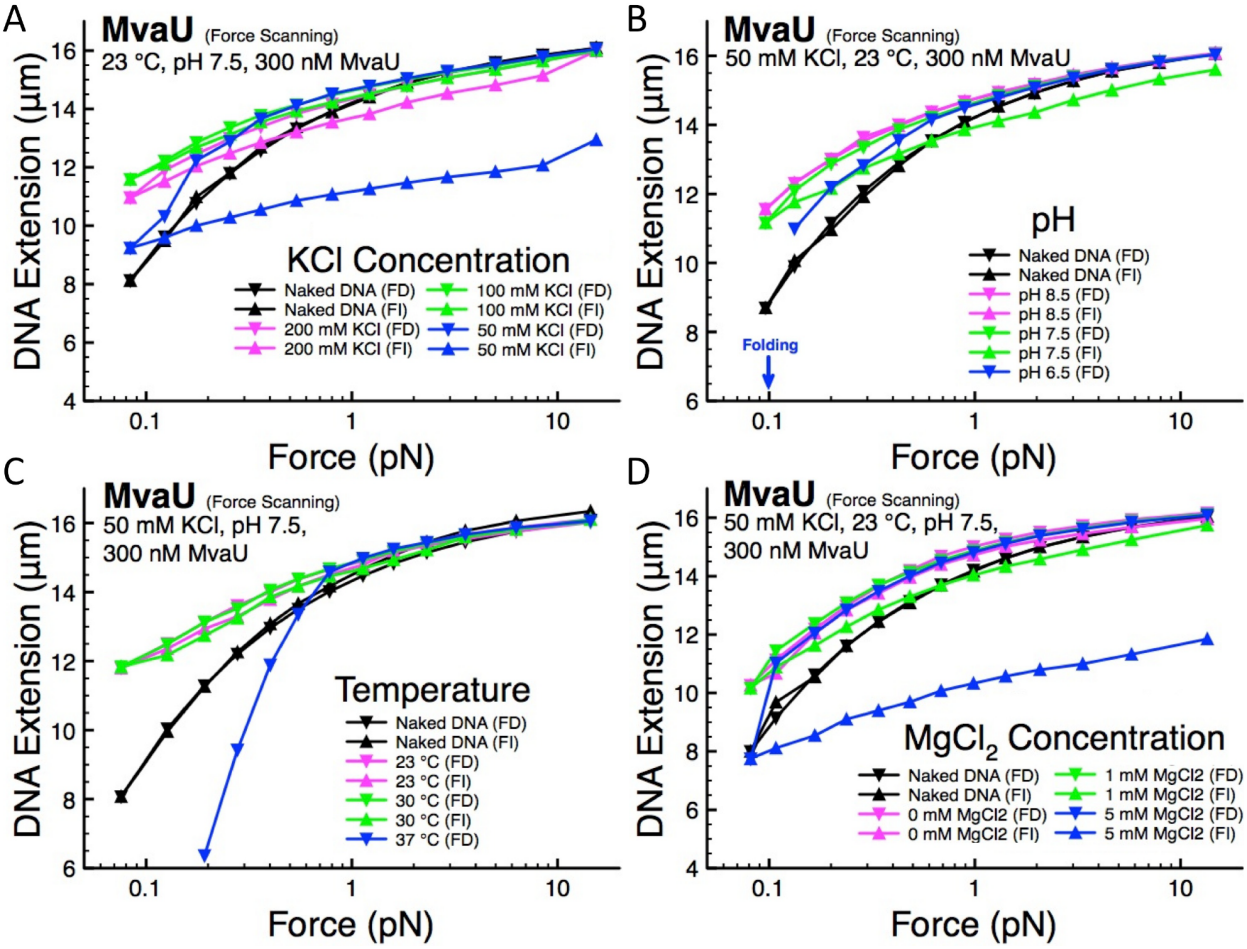
The effect of variation in KCl concentration, pH, temperature, and MgCl_2_ concentration on MvaU-DNA. The concentration of MvaU was fixed at 300 nM MvaU at all the conditions tested. (A) Lower KCl concentration results in larger hysteresis or DNA folding. The apparent DNA stiffening is largely unaffected in 50–200 mM KCl. (B) Lower pH value results in larger hysteresis or DNA folding, while the level of DNA stiffening is largely unaffected in the pH range tested. (C) Increase in temperature leads to larger hysteresis or DNA folding, while the level of DNA stiffening is largely unaffected in the temperature range tested. (D) In the presence of MgCl_2_, DNA folding is promoted as indicated by increasing amount of hysteresis as the MgCl_2_ concentration was increased. The level of DNA stiffening is largely unaffected by variation in MgCl_2_ concentration.

In 200 mM KCl, MvaU stiffens the DNA with slight hysteresis between the force-decrease and force-increase curves (Figure 4A, magenta data). Maintaining the same MvaU concentration of 300 nM, we reduced the KCl concentration to 100 mM, resulting in a similar level of DNA stiffening and hysteresis (Figure 4A, green data). In 50 mM KCl, we still found similar level of DNA stiffening at forces >1 pN in the force-decrease curve, but hysteresis is significantly increased, indicating moderate DNA folding (Figure 4A, blue data). Note that at forces <0.3 pN in the force-decrease curve, the reduction in extension is due to MvaU-induced DNA folding that predominates at lower forces. These results suggest that the level of DNA stiffening caused by MvaU binding is not significantly affected by variation in KCl concentration, as force-decrease curves at forces >1 pN are largely unaffected in the range of KCl concentration tested (Figure 4A). However, the level of hysteresis between force-decrease and force-increase curves increases as KCl concentration was decreased, indicating that MvaU-induced DNA folding increases at lower KCl concentration. This indicates more folding over our experimental time scale, but it does not imply that DNA folding has reached equilibrium. The DNA folding promoted at lower KCl concentration is anticipated due to the increased electrostatic attraction between MvaU and DNA. As the osmolarity of bacteria can vary widely depending on the environment, our results suggest that MvaU can affect chromosomal DNA organization.

Similar experiments were carried out to study the effect of variation in pH, temperature, and MgCl_2_ concentration within their respective physiological ranges. We examined the force-extension curves of MvaU-DNA in pH range of 6.5–8.5 (Figure 4B), temperature range of 23–37°C (Figure 4C), and MgCl_2_ concentration range of 0–5 mM (Figure 4D). The results suggest that variation in these factors do not significantly affect the level of DNA stiffening, while DNA folding is facilitated at lower pH, higher temperature, or higher concentrations of MgCl_2_. Although the level of hysteresis varies from one experiment to another, the general trend of increased or decreased level of DNA folding in response to variation in solution conditions is reproducible and consistent across experiments (see Figure S1C–F for additional independent experiments).

Taken together, our results show that MvaU nucleoprotein filament stiffness is largely insensitive to variation in KCl concentration, pH, temperature, and MgCl_2_ concentration over the ranges tested. MvaU-induced DNA folding, however, is moderately regulated by these environmental factors.

### MvaU nucleoprotein filament can protect DNA from DNase1 cleavage

It has been reported MvaU can also repress phase-variable expression of MvaT-regulated *cupA* gene cluster *in* *vivo*, and it is found able to complement the phenotypes of an MvaT-deficient strain [22]. It is therefore likely that both MvaT and MvaU function coordinately as gene repressor through their DNA binding [20]. Recently, the formation of rigid nucleoprotein filaments by StpA in *E. coli* and by Lsr2 in Gram-positive bacteria *Mycobacterium tuberculosis* has been found to restrict DNA accessibility [34], [35]. This DNA blocking capability is proposed as the mechanism responsible for their function as gene silencers. Here we would like to examine whether MvaU can also block DNA accessibility.

**Figure 5:**
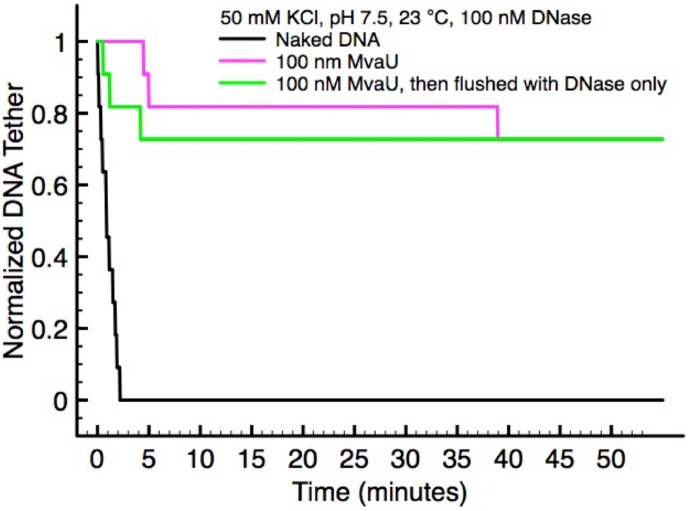
The formation of MvaU nucleoprotein filament effectively blocks DNase1 access to DNA. Multiple tethers of λ-DNA were completely cleaved in the presence of 100 nM DNase1 after <5 minutes of incubation (black data). In contrast, ∼70% of DNA tethers remained uncut ∼1 hour after the mixture of MvaU/DNase1 was introduced (magenta data), or after the buffer was changed to those containing 100 nM DNase1 without free MvaU in solution (green data).

We performed a single-molecule DNase1 cleavage assay to study DNA accessibility of MvaU-DNA to cleavage by DNase1 as described previously [34]. Multiple DNA tethers were stretched in the focal plane at ∼7 pN to prevent folding and solution containing DNase1 was then introduced. DNA tethers were progressively cleaved, indicated by loss of detected DNA tethers (Figure 5, black data). The DNA tethers were completely cleaved by DNase1 in <5 minutes after solution containing 100 nM DNase1 in 50 mM KCl, pH 7.5, at 23°C was introduced. The rate of DNA cleavage can give us valuable information on DNA accessibility. In another independent experiment, we incubated the DNA tethers with 100 nM MvaU for 5 minutes to allow the formation of MvaU nucleoprotein filaments. Following this, solution containing a mixture of 100 nM MvaU and 100 nM DNase1 was introduced. We found that the formation of MvaU nucleoprotein filament and/or the presence of 100 nM MvaU in solution can significantly reduce the rate of cleavage by DNase1, as ∼70% of DNA tethers were still intact for ∼1 hour after DNase1 was introduced (Figure 5, magenta data).

The above DNase1 cutting experiment was conducted in the presence of 100 nM MvaU, which can also reduce the cleavage rate through competitive DNA binding. However, we speculate that the protection from DNase1 cleavage is caused by the formation of rigid nucleoprotein filaments. To test this hypothesis, we incubated the DNA tethers with 100 nM MvaU for 5 minutes to allow the formation of MvaU nucleoprotein filaments. Afterwards, we introduced solution containing 100 nM DNase1 only. Due to the absence of MvaU protein in solution, any reduction in cleavage rate can only arise from the prebound MvaU on DNA. We found that ∼70% of DNA tethers were still intact for ∼1 hour after solution containing 100 nM DNase1 was introduced (Figure 5, green data). Note that the protection from DNase1 cleavage is largely caused by formation of MvaU nucleoprotein filaments, as a force of ∼7 pN was applied on the DNA tethers throughout the experiments to minimize DNA bridging or compaction. Under these conditions, MvaU forms rigid nucleoprotein filaments, which are remarkably stable as they do not dissociate from DNA even after ∼1 hour in the absence of free MvaU protein. In the presence of DNA bridging or folding, we expect an even higher level of protection since folding may further reduce the exposed DNA site.

Overall, our single-molecule DNase1 cleavage assay reveals that the formation of MvaU nucleoprotein filaments can effectively block DNA accessibility. This finding further highlights the potential physiological importance of filament formation for their gene-silencing function.

## Discussion

### The organization modes of MvaU to DNA

In this work, we have elucidated the DNA organization modes of MvaU using magnetic tweezers and AFM. Our magnetic tweezers experiments revealed that MvaU can stiffen DNA and cause DNA folding at high MvaU concentrations. These results are complemented by AFM imaging experiments, which revealed the various conformations of MvaU-DNA, such as DNA bridges, MvaU nucleoprotein filaments, and compact DNA structures. The DNA stiffening found in our stretching experiments is caused by the formation of rigid MvaU nucleoprotein filaments, while the apparent DNA folding corresponds to the compact DNA structures found in the imaging experiments. Importantly, we also found that these two mechanisms are not contradictory to each other, as DNA stiffening can occur prior to DNA folding (Figure 2A, blue data). In support of this, our imaging experiments show that MvaU nucleoprotein filaments can be further compacted into thicker filaments (Figure 3C–D). We use non-specific DNA molecules in our experiments, as MvaU binds to hundreds of distinct genomic regions and we expect that the DNA organization mode of MvaU to be independent to DNA sequences [20].

The formation of DNA bridges at lower stoichiometry of 1 MvaU monomer to 10 bp suggests that MvaU can associate different segments of naked DNA molecules (Figure 3B). In addition, two or more protein-coated DNA molecules can be further compacted to form higher-order rod-like structures (Figure 3C–D). MvaT and MvaU can oligomerize and form homomeric and heteromeric complexes through their N-terminal regions [22]. Therefore, we speculate that similar to MvaT, the formation of compact structures at higher MvaU concentration is caused by concentration-dependent high-order oligomerization of MvaU in solution [22], [32], [33], [40]. Different oligomeric conformations may correspond to different DNA binding properties. Similar to H-NS, we propose that once a DNA site is bound by an MvaU binding unit, more MvaU will oligomerize across adjacent DNA stretch, forming nucleoprotein filaments [43]. Further, MvaU may possess an additional low-affinity oligomerization domain that can mediate higher-order compaction of these nucleoprotein filaments at sufficiently high concentration.

### The implication of MvaU binding on its functional role

MvaU serves as a transcriptional silencer for some genes, such as those for prophage activation, pyocyanin synthesis, fimbrial *cupA* gene expression, formation of lectin and other exoproducts, and many others [18], [40]. Previously, the formation of nucleoprotein filaments has been reported for H-NS, StpA, MvaT, and Lsr2, all of which function as gene repressors [32], [34], [35], [41]. It has been proposed that nucleoprotein filament formation may serve as the basis for gene silencing, and possibly a general gene silencing mechanism across prokaryotes [28]. In this work, we found that MvaU, which can perform some of MvaT’s regulatory functions, can also form rigid nucleoprotein filaments that give rise to DNA stiffening in our stretching experiments. We also found that the formation of rigid MvaU nucleoprotein filaments restricts DNA accessibility, suggesting that the genetic information contained in the DNA is deemed inaccessible to transcription by RNA polymerase. This way, gene-silencing function can be achieved.

In addition to filament formation, we also found that MvaU can mediate high-order DNA organization at high protein concentration. Further, our data suggests that multiple environmental factors, such as KCl concentration, pH, temperature, and MgCl_2_ concentration, can affect the level of DNA folding over physiologically relevant range. To our knowledge, there is no report on the potential role of MvaU on chromosomal DNA packaging. Our finding suggests that MvaU can effectively condense DNA and may play a role in packaging the bacterial nucleoid.

### Comparison between MvaT and MvaU organization modes and their roles *in* *vivo*

In *Pseudomonas aeruginosa*, MvaU is a transcription regulator that shares 47% amino acid identity and 65% similarity with MvaT [22]. The resemblance of MvaT and MvaU goes beyond their structure and function, as we found striking similarities in their DNA organization modes. Both MvaT and MvaU can form rigid nucleoprotein filaments that can block access to DNA, and they can organize DNA into more complex compact structures at higher protein concentration. The nucleoprotein filaments formed by MvaT and MvaU are generally insensitive to variation in KCl concentration, pH, temperature, and MgCl_2_ concentration, while the level of DNA folding is moderately modulated by these factors over physiological ranges [32].

The similarities in DNA organization modes of MvaT and MvaU may correspond to their predicted functional redundancy *in* *vivo*. In terms of DNA binding, MvaT and MvaU have nearly complete binding profile overlap in the genome, showing that they control the expression of largely the same set of genes [20]. The loss of gene encoding MvaT or MvaU leads to increase in production of the other, showing the reciprocity and cross-regulation of these two proteins [22]. The loss of both proteins, however, cannot be tolerated and causes profound effects on the cell [20], [40], [44]. Based on our previous work on MvaT and our current findings on MvaU, we conclude that MvaT and MvaU bind to DNA in a largely similar manner, corresponding to their *in* *vivo* reciprocity. They can form gene-silencing nucleoprotein filaments to regulate gene expression, and are possibly involved in chromosomal DNA packaging. The existence of multiple H-NS paralogues in the same microorganism of *Pseudomonas* genus may be advantageous and serve to maintain a functional gene regulatory system, and therefore may be more than to simply serve as a backup system [5], [20].

Finally, there are many other multiple H-NS paralogues that exist in the same microorganism [14]. It remains unclear how these seemingly redundant proteins can pose evolutionary benefits to the bacterial cell. It has been suggested that the existence of more than one H-NS-related protein may correspond to functional divergence and partition [14]. It is also possible that multiple H-NS paralogues integrate multiple environmental signals to promote higher adaptability, notably the *Pseudomonas* genus that exhibits remarkable ecological and metabolic diversity [14], [45]. In addition, the ability of these H-NS paralogues to form heteromeric complexes *in* *vivo* through their oligomerization domain may pose additional functional significance. As MvaU bears similar DNA organization modes to MvaT, we expect that MvaT-MvaU heteromeric complexes will also exhibit similar DNA binding properties compared to its constituents. To test this, we complexed MvaT and MvaU in a test tube with ∼10 mM concentration for each protein at room temperature for 4 hours to allow the two proteins to associate and reach equilibrium distribution of homomeric and heteromeric complexes. Although the formation of heteromeric complexes cannot be directly confirmed in our experiments, we reason that heteromeric complexes should predominate in the mixture as MvaT copurifies with MvaU [18], [22]. Following this, we examined the DNA binding of MvaT-MvaU mixture with single-DNA stretching experiments, and we found that the mixture has similar DNA binding properties compared to MvaT and MvaU (Figure S3). The results of our studies on MvaT and MvaU give insights and advance the understanding on the nature on such intriguing bacterial regulatory system.

## Supporting Information

Figure S1 **Single molecule stretching of MvaU-DNA complexes.** Additional independent experiments were shown to demonstrate the repeatability of the trends and consistency of the data. Note the variation on the level of hysteresis compared to the data in the main text at the same condition due to the non-equilibrium nature of DNA folding.
(TIF)

Figure S2 **The effect of variation in KCl concentration, pH, temperature, and MgCl_2_ concentration on naked DNA.** Naked DNA shows negligible variation in its elasticity when we varied KCl concentration (A), pH (B), temperature (C), and MgCl_2_ concentration (D). Hence, any observed changes in force-extension curves in the presence of protein due to variation in these factors can be attributed to changes in protein-DNA interaction.
(TIF)

Figure S3 **Single-molecule stretching experiments on DNA complexed with MvaT-MvaU mixture.** (A) Increasing concentration of MvaT-MvaU mixture leads to DNA stiffening, accompanied by increasing amount of DNA folding as indicated by hysteresis. (B) Time-course data of the stretching experiment in panel A, showing progressive DNA folding as force is decreased, followed by unfolding of the protein-DNA complexes as force is increased.
(TIF)
