# Supplementary figures and images for: Single-Molecule Study on Histone-Like Nucleoid-Structuring Protein (H-NS) Paralogue in Pseudomonas aeruginosa: MvaU Bears DNA Organization Mode Similarities to MvaT
Source: PLoS One. 2014 Nov 5;9(11):e112246. doi: 10.1371/journal.pone.0112246 (PMC4221281; doi:10.1371/journal.pone.0112246)

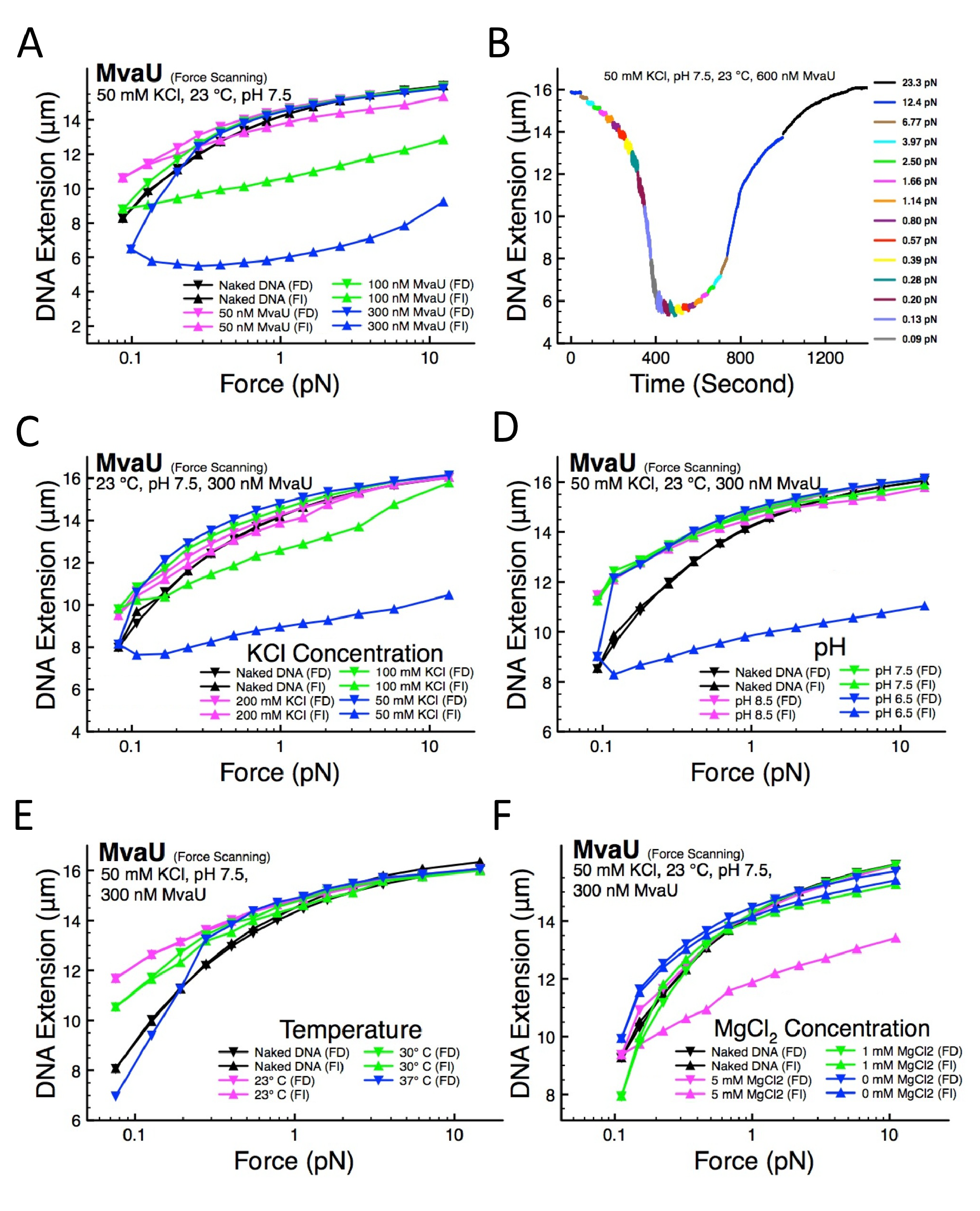

Supplement: Figure S1 — Single molecule stretching of MvaU-DNA complexes. Additional independent experiments were shown to demonstrate the repeatability of the trends and consistency of the data. Note the variation on the level of hysteresis compared to the data in the main text at the same condition due to the non-equilibrium nature of DNA folding. (TIF) [file pone.0112246.s001.tif]

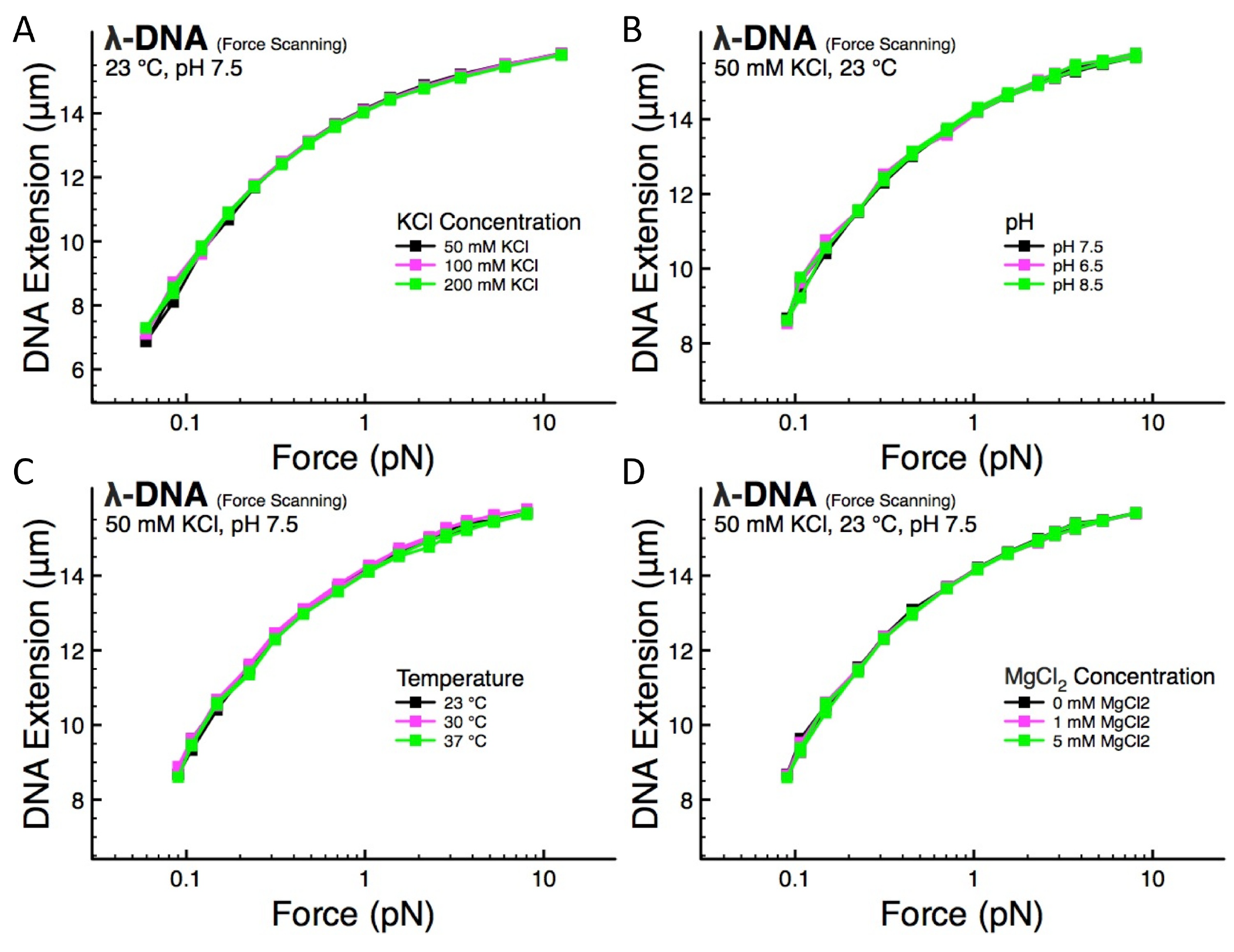

Supplement: Figure S2 — The effect of variation in KCl concentration, pH, temperature, and MgCl2 concentration on naked DNA. Naked DNA shows negligible variation in its elasticity when we varied KCl concentration (A), pH (B), temperature (C), and MgCl2 concentration (D). Hence, any observed changes in force-extension curves in the presence of protein due to variation in these factors can be attributed to changes in protein-DNA interaction. (TIF) [file pone.0112246.s002.tif]

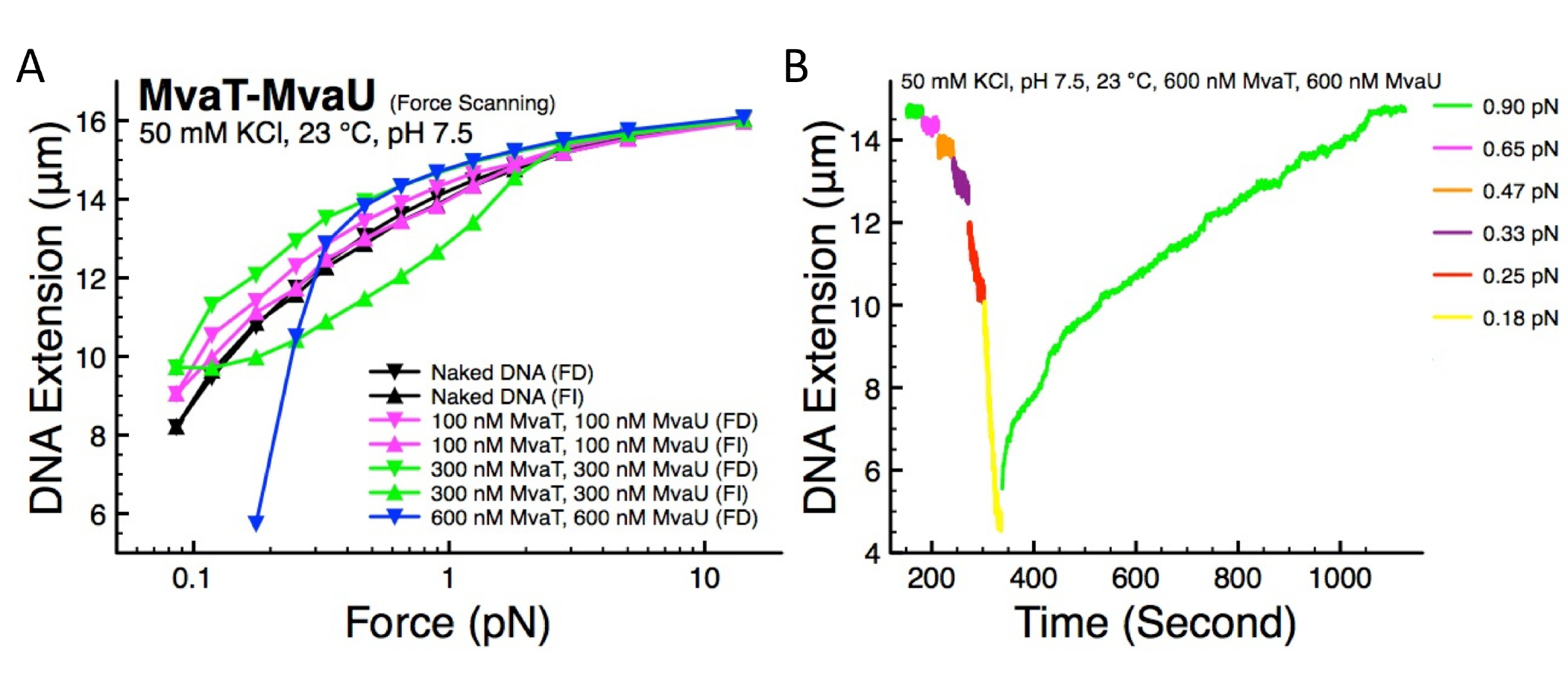

Supplement: Figure S3 — Single-molecule stretching experiments on DNA complexed with MvaT-MvaU mixture. (A) Increasing concentration of MvaT-MvaU mixture leads to DNA stiffening, accompanied by increasing amount of DNA folding as indicated by hysteresis. (B) Time-course data of the stretching experiment in panel A, showing progressive DNA folding as force is decreased, followed by unfolding of the protein-DNA complexes as force is increased. (TIF) [file pone.0112246.s003.tif]
